# LRCS: Duality, LP bounds, and field size

**DOI:** 10.1007/s10623-026-01829-7

**Published:** 2026-04-25

**Authors:** Anina Gruica, Benjamin Jany, Alberto Ravagnani

**Affiliations:** 1 Technical University of Denmark, Kongens Lyngby, Denmark; 2 Department of Mathematics and Computer Science, Eindhoven University of Technology, Eindhoven, The Netherlands

**Keywords:** Error-correcting codes, Distributed storage, Locally recoverable codes, LP bound

## Abstract

We develop a duality theory of locally recoverable codes (LRCs) and apply it to establish a series of new bounds on their parameters. We introduce and study a refined notion of weight distribution that captures the code’s locality. Using a duality result analogous to a MacWilliams identity, we then derive an LP-type bound that improves on the best known bounds in several instances. Using a dual distance bound and the theory of generalized weights, we obtain non-existence results for optimal LRCs over small fields. In particular, we show that an optimal LRC must have both minimum distance and block length relatively small compared to the field size.

## Introduction

In the last decade, *locally recoverable codes* have been a central topic in communication and distributed storage [5, 9, 12, 17, 31]. A code has good locality properties when each coordinate of each codeword can be recovered from a small set of other entries of the same codeword. This property is captured by a parameter of the underlying code called *locality*. Small locality allows, for instance, a fast recovery process when the code is used for distributed storage. Ideally, a code has both small locality and large minimum distance, as the latter offers protection from errors and erasures.

Most of the research on locally recoverable codes focuses on bounds [1, 5, 12, 15, 17], constructions and decoding [3, 4, 6, 7, 10, 21, 22, 24, 31]. This paper pertains to the former line of research.

As one expects, a code cannot have all the desirable properties at the same time. In particular, it cannot have arbitrarily small locality while having large minimum distance for the given dimension and block length. The trade-offs among all these parameters are captured by various bounds, the best known of which is probably the *Singleton-type Bound* [12]. Codes attaining this bound with equality are known to exist for some parameter sets over large fields. Over small fields, and to the best of our knowledge, some constructions are known specific to the size of the underlying field (for example, see [28, 34] for $q=4$). Generally it is still an open question for which parameter sets locally recoverable codes can exist.

In this paper, we investigate two aspects of the theory of locally recoverable codes: their *duality theory* and *field size*. Part of this work was presented and published at the Information Theory Workshop (ITW) in 2023; see [14].

We first demonstrate that duality results, besides being mathematically interesting, represent a very powerful tool for investigating the parameters of locally recoverable codes and establishing new bounds. In the second part of the paper we derive various results that link the size of the underlying field to the other parameters of a locally recoverable code. In particular, we prove that codes attaining the Singleton-type bound of [12] cannot exist for some parameter sets.

The rest of the introduction briefly illustrates the contributions made by this paper, pointing the reader to the relevant sections.

After recalling the basics of locally recoverable codes in Sect. 2, we introduce a refined notion of weight distribution of a code, which is able to capture its locality as well as the weights of the codewords; see Sect. 3. In the same section, we then establish a duality result for the refined weight distribution, which is similar to, and yet different from, a MacWilliams identity. The identity combined with linear programming produces a bound on the parameters of a locally recoverable code that improves the best bounds currently available for several parameter sets; see Sect. 4. We illustrate this with comparison tables and commenting about how results relate to the state of the art, including other LP-type bounds.

The last part of the paper, namely Sect. 5, is devoted to the role played by the field size in the theory of locally recoverable codes. We propose various arguments based on the notions of dual distance and generalized weights. This leads us to establishing new bounds for the parameters of a locally recoverable code, which in turn gives us non-existence results for codes meeting the Singleton-type Bound over small fields.

## Locally recoverable codes

We start by recalling concepts of classical coding theory and locally recoverable codes, and by establishing the notation. Throughout the paper, $n \ge 2$ is an integer, *q* denotes a prime power, and $\mathbb {F}_q$ is the finite field of *q* elements. We will denote the set $\{1, \dots , n\}$ by [*n*].

### Definition 2.1

A (**linear**) **code** is an $\mathbb {F}_q$-linear subspace $\mathscr {C}\le \mathbb {F}_q^n$ endowed with the Hamming metric. The **dual** of $\mathscr {C}$ is $\mathscr {C}^\perp :=\{x \in \mathbb {F}_q^n: x\cdot y^\top = 0 \text { for all } y \in \mathscr {C}\}.$ Moreover, we say that $\mathscr {C}\le \mathbb {F}_q^n$ is **non-degenerate** if there is no $i \in [n]$ for which each $x \in \mathscr {C}$ has $x_i=0$. The codes $\{0\}$ and $\mathbb {F}_q^n$ are called **trivial**.

In this document, by “code” we always mean “non-trivial code”, unless otherwise specified.

### Definition 2.2

The **minimum distance** of a code $\mathscr {C}$ is defined as

$$\begin{aligned} d(\mathscr {C}) := \min \{\omega ^{\text {H}}(x) : x \in \mathscr {C}\setminus \{0\}\}, \end{aligned}$$

where $\omega ^{\text {H}}(x):=|\{i : \, x_i \ne 0\}|$ is the (**Hamming**) **weight** of *x*.

A large minimum distance guarantees the error detection and correction capabilities of a code, so naturally one wants this parameter to be as large as possible. However, as it is well known, there is a trade-off between the minimum distance and the dimension of a code with given length. This trade-off is expressed by the famous *Singleton Bound*, which says that for a code $\mathscr {C}\le \mathbb {F}_q^n$ we always have $d(\mathscr {C}) \le n - \dim _{\mathbb {F}_q}(\mathscr {C}) +1$; see [30]. Codes that meet the bound with equality are called **MDS**. It remains a wide open problem in coding theory to determine when codes achieving this bound (or other bounds) exist.

Apart from the minimum distance and the dimension, in this paper we consider an additional parameter for linear codes, called *locality*.

### Definition 2.3

A code $\mathscr {C}\le \mathbb {F}_q^n$ has **locality** *r* if for every $i \in \{1, \ldots , n\}$ there exists a set $S_i \subseteq [n]$ with the following properties:

1. $$i \notin S_i$$,
2. $$|S_i| \le r$$,
3. if $x, y \in \mathscr {C}$ and $x_j = y_j$ for all $j \in S_i$, then $x_i = y_i$.

We call **LRC** (**locally recoverable code**) a code for which the locality parameter is considered. A set $S_i$ satisfying 3) is called a **recovery sets** for (the coordinate) *i*.

### Example 2.4

Let $\mathscr {C}\le \mathbb {F}_2^7$ be the binary simplex code of dimension 3, which consists of codewords of the form

$$\begin{aligned} (u_1,u_2,u_3)\begin{pmatrix} 1 &  0 &  0 &  1 &  0 &  1 &  1 \\ 0 &  1 &  0 &  1 &  1 &  0 &  1 \\ 0 &  0 &  1 &  0 &  1 &  1 &  1 \end{pmatrix} = (u_1,u_2,u_3,u_1+u_2,u_2+u_3,u_1+u_3,u_1+u_2+u_3), \end{aligned}$$

where $u_1,u_2,u_3 \in \mathbb {F}_2$. Without loss of generality, we focus on recovering coordinate $i=1$. It is not hard to check that recovery sets for 1 are

$$\begin{aligned} \{2,4\},\, \{3,6\},\, \{5,7\},\, \{2,3,7\},\, \{4,6,7\},\, \{3,4,5\},\, \{2,5,6\} \end{aligned}$$

as well as any subset of $\{2,\dots ,7\}$ containing one of the above. By symmetry of the coordinates, this code has (minimum) locality 2.

As the name suggests, a recovery set $S_i$ for *i* allows to recover the coordinate $x_i$ of any codeword $x \in \mathscr {C}$ using only the coordinates of *x* indexed by $S_i$. This is done via a **recovery function** $f_i: \pi _{S_i}(\mathscr {C}) \rightarrow \mathbb {F}_q$ that satisfies $f_i(\pi _{S_i}(x)) = x_i$ for all $x \in \mathscr {C}$, where $\pi _{S_i}$ denotes the projection map onto the coordinates indexed by the elements of $S_i$. Interestingly, for linear codes the recovery functions must be linear; see [1, Lemma 10].

### Proposition 2.5

Let $\mathscr {C}\le \mathbb {F}_q^n$ be a code and let $S_i$ be a recovery set for the coordinate *i* with recovery function $f_i$. Then $f_i$ is an $\mathbb {F}_q$-linear map.

Similarly to classical codes, it is important to understand the trade-offs between the locality parameter and other parameters of the code, such as its minimum distance and dimension. In [12], the authors showed how locality impacts the dimension of an LRC by establishing a generalization of the Singleton Bound.

### Theorem 2.6

(Singleton-Type Bound) Let $\mathscr {C}\le \mathbb {F}_q^n$ be a code with locality *r*, dimension *k*, and minimum distance *d*. Then

2.1 $$\begin{aligned} k + \left\lceil \llceil \frac{k}{r} \right\rceil \rrceil \le n - d+2. \end{aligned}$$

Note that the bound of Theorem 2.6 coincides with the classical Singleton Bound if $k=r$. Codes whose parameters meet the bound (2.1) with equality are called **optimal LRC**. Note that the simplex code in Example 2.4 is optimal.

The most celebrated construction of optimal LRC is for $q \ge n$, $r \mid k$, and $r+1 \mid n$; see [31]. Constructions involving different constraints on the parameters also exist, but still require the field size to be large enough (e.g. see [22]). However, in general optimal LRC do not always exist or may not have been found yet. In order to establish or exclude the existence of optimal LRCs for small *q*, it is natural to establish bounds linking the locality to the size of the underlying field. The following is a shortening bound established in [5], that improves the Singleton-type bound in Theorem 2.6. In the minimum we include the (trivial) case $t=0$, even though the original statement does not. Note that for some parameters the minimum is indeed attained by $t=0$.

### Theorem 2.7

(Shortening Bound) Let $\mathscr {C}\le \mathbb {F}_q^n$ be a code with locality *r*, dimension *k* and minimum distance *d*. We have

2.2 $$\begin{aligned} k \le \min _{t \in \mathbb {Z}_{\ge 0}}\left\{ rt+k_{\text {opt}}^{(q)}(n-t(r+1),d)\right\} , \end{aligned}$$

where $\smash {k_{\text {opt}}^{(q)}(n,d)}$ is the largest possible dimension of a code of length *n* and minimum distance *d* over $\mathbb {F}_q$.

### Remark 2.8

(i) It was observed in [17] that (2.2) yields a series of bounds on codes with locality, by applying known bounds on $\smash {k_{\text {opt}}^{(q)}(n-t(r+1),d)}$. For example, by letting $\smash {t = \left\lceil \llceil k/r \right\rceil \rrceil }$ and using the classical Singleton Bound on $\smash {k_{\text {opt}}^{(q)}(n-t(r+1),d)}$, we recover Theorem 2.6.
(ii) Even though the bound of Theorem 2.7 is a refinement of Theorem 2.6, there is a “computational” drawback when evaluating it. Indeed, determining the value of $k_{\text {opt}}^{(q)}(n,d)$ for given *d*, *n*, *q* is a wide open problem in classical coding theory, which means that no closed formula is known for the RHS of (2.2). Establishing bounds whose evaluation is computationally feasible has therefore become a crucial research problem in the study of LRCs. This is one of the problems this paper addresses.

The concept of locally recoverable codes was further generalized in [25], where the authors introduce the notion of an $(r, \delta )$-LRC. The additional parameter $\delta$ provides extra information regarding the number of recovery sets for coordinate *i* of size less than or equal to *r*. In the applications, $(r, \delta )$-LRCs facilitate the local recovery of a failed node in the event that other nodes of the network fail as well.

### Definition 2.9

A non-degenerate code $\mathscr {C}\le \mathbb {F}_q^n$ has **locality** $(r, \delta )$ (or is an $(r,\delta )$-**LRC**) if for all $i \in [n]$ there exists a set $S_i \subseteq [n]$ such that:

1. $$i \notin S_i$$,
2. $$|S_i| \le r+\delta -2$$,
3. $$d(\pi _{S_i \cup \{i\}}(\mathscr {C})) \ge \delta $$.

We then call $S_i$ an $(r,\delta )$-**recovery set** for *i*.

Note that for an $(r,\delta )$-LRC $\mathscr {C}\le \mathbb {F}_q^n$, an $(r,\delta )$-recovery set $S_i$ for $i \in [n]$, and $T \subseteq S_i \cup \{i\}$ with $|T|=\delta -1$, the coordinates of any $x \in \mathscr {C}$ indexed by *T* can be recovered from the coordinates of *x* indexed by $\left( S_i\cup \{i\} \right) \setminus T$. This follows from Definition 2.9, part 3). Moreover, it can easily be seen that the notions of $(r,\delta )$-LRC and LRC with locality *r* coincide when $\delta =2$.

A refinement of Theorem 2.6 taking the parameter $\delta$ into account was established in [25] and it reads as follows:

2.3 $$\begin{aligned} d \le n - k +1 - \left( \left\lceil \llceil \frac{k}{r} \right\rceil \rrceil -1\right) (\delta -1). \end{aligned}$$

Furthermore, the following is a generalization of Theorem 2.7 which was established in [13, 27]:

2.4 $$\begin{aligned} k \le \min _{t \in \mathbb {Z}_{\ge 0}} \{tr + k_{\text {opt}}^{(q)}(n-t(r+\delta -1),d)\}. \end{aligned}$$

Similarly to Theorem 2.7, the bound of (2.4) has a computational drawback, as already explained in Remark 2.8.

Note that both (2.1) and (2.2) can be recovered by the two previous bounds when $\delta = 2$.

In this paper, we focus mostly on classical LRCs (i.e. $\delta =2)$. However, in some instances we consider the broader case of $(r, \delta )$-LRCs for any $\delta$, when the techniques we develop are applicable.

## A finer weight distribution and duality

In this section, we introduce and work with a refinement of the classical *weight distribution* of a code. Recall that the **weight distribution** of $\mathscr {C}\le \mathbb {F}_q^n$ is the tuple $(W_0(\mathscr {C}), \dots , W_n(\mathscr {C}))$, where

$$\begin{aligned} W_i(\mathscr {C}) := |\{x \in \mathscr {C}: \omega ^{\text {H}}(x) = i\}|. \end{aligned}$$

Before we introduce the aforementioned refinement of the weight distribution, we show how the locality of a code $\mathscr {C}\le \mathbb {F}_q^n$ can be captured by looking at the support of codewords in the dual code $\mathscr {C}^{\perp }$. Recall that for a vector $x \in \mathbb {F}_q^n$, its **support** is defined to be the set $\sigma (x):= \{ i \mid x_i \ne 0\}$. We now have the following equivalent characterization of the locality parameter, which will be used extensively in this paper.

### Lemma 3.1

Let $r \ge 1$ be an integer. A linear code $\mathscr {C}\le \mathbb {F}_q^n$ has locality *r* if and only if for any $i \in [n]$ there exists $x \in \mathscr {C}^{\perp }$ with $i \in \sigma (x)$ and $\omega ^{\text {H}}(x) \le r+1$.

Lemma 3.1 was first established in [15, Lemma 5] and heavily relies on the fact that recovery functions for linear codes are always linear; see Proposition 2.5. The characterization of Lemma 3.1 inspires the following definition, where instead of caring only about how many codewords of a certain weight there are (which is captured in the classical weight distribution), we want to know the number of codewords of a certain weight having a certain subset of [*n*] in their support.

### Definition 3.2

For a code $\mathscr {C}\le \mathbb {F}_q^n$, a set $S \subseteq [n]$, and $0 \le i \le n$, we let

$$\begin{aligned} W_i^S(\mathscr {C}) := |\{x \in \mathscr {C}: \omega ^{\text {H}}(x) = i, \, S \subseteq \sigma (x)\}|. \end{aligned}$$

Note that if we set $S=\emptyset$, then the refined weight distribution introduced in Definition 3.2 recovers the classical weight distribution. The main result of this section is a MacWilliams-type identity for the refined weight distribution. In more detail, we will show that the refined weight distribution of the dual code fully determines the refined weight distribution of the original code.

We start with the following result, which relates the values of the refined weight distributions to one another.

### Lemma 3.3

Let $\mathscr {C}\le \mathbb {F}_q^n$ and $A \subseteq [n]$. Then for all $1 \le i \le n$ and $|A| \le t \le i$ we have

$$\begin{aligned}W_i^A(\mathscr {C}) = \frac{1}{{\left( {\begin{array}{c}i-|A|\\ t- |A|\end{array}}\right) }}\sum _{\begin{array}{c} A \subseteq S \subseteq [n] \\ |S| = t \end{array}^{\,}} W_i^S(\mathscr {C}).\end{aligned}$$

### Proof

Let $\mathscr {V}= \{ (x, S) \mid x \in \mathscr {C}, \, \omega ^{\text {H}}(x) = i, \, A \subseteq S \subseteq \sigma (x), \, |S| = t\}$. We count the elements of $\mathscr {V}$ in two ways. On one hand, we have

3.1 $$\begin{aligned} |\mathscr {V}|= \sum _{\begin{array}{c} A \subseteq S \subseteq [n] \\ |S| = t \end{array}^{\,}} W_i^S(\mathscr {C}). \end{aligned}$$

On the other hand,

3.2 $$\begin{aligned} |\mathscr {V}| = \sum _{\begin{array}{c} x \in \mathscr {C}\\ \omega ^{\text {H}}(x) = i \\ A \subseteq \sigma (x) \end{array}}| \{S \, : \, A \subseteq S \subseteq \sigma (x) and |S| = t\}| ={i -|A| \atopwithdelims ()t - |A|} W_i^A(\mathscr {C}). \end{aligned}$$

Combining (3.1) and (3.2) concludes the proof. $\square$

In order to obtain the MacWilliams-type identity for the refined weight distribution from Definition 3.2, we need to introduce auxiliary definitions and results.

### Definition 3.4

For a code $\mathscr {C}\le \mathbb {F}_q^n$ and subsets $S \subseteq T \subseteq [n]$, let

$$\begin{aligned} \mathscr {C}(S,T):=\{x \in \mathscr {C}: S \subseteq \sigma (x) \subseteq T\}. \end{aligned}$$

Moreover, $\mathscr {C}(T)$ denotes the **shortening** of $\mathscr {C}$ by the set *T*, i.e., $\mathscr {C}(T)=\{x \in \mathscr {C}: \sigma (x) \subseteq T\}$.

We can now express the cardinality of a subcode introduced in Definition 3.4 in terms of the cardinality of subcodes of the dual code.

### Proposition 3.5

Let $\mathscr {C}\le \mathbb {F}_q^n$. For all $S \subseteq T \subseteq [n]$ we have

$$\begin{aligned} |\mathscr {C}(S,T)| = |\mathscr {C}| \sum _{A \subseteq S} (-1)^{|A|}\displaystyle \frac{|\mathscr {C}^\perp (T^c \cup A)|}{q^{n-|T|+|A|}}. \end{aligned}$$

### Proof

We have

3.3 $$\begin{aligned} |\mathscr {C}(S,T)|&= |\mathscr {C}(T)|- |\{x \in \mathscr {C}: \sigma (x) \subseteq T, \, \sigma (x) \subseteq [n]\setminus A \text { for some }\, \emptyset \ne A \subseteq S\}| \nonumber \\&= |\mathscr {C}(T)|- |\{x \in \mathscr {C}: \sigma (x) \subseteq T \cap ([n]\setminus A) \text { for some}\, \emptyset \ne A \subseteq S\}|. \end{aligned}$$

Since $A \subseteq S \subseteq T$, we can rewrite (3.3) as

$$\begin{aligned} |\mathscr {C}(T)|- |\{x \in \mathscr {C}: \sigma (x) \subseteq T\setminus A \text { for some}\, \emptyset \ne A \subseteq S\}|&= |\mathscr {C}(T)|- \left| \bigcup _{\emptyset \ne A \subseteq S} \mathscr {C}(T \setminus A)\right| \\&= \sum _{A \subseteq S} (-1)^{|A|}\left| \mathscr {C}(T \setminus A)\right| , \end{aligned}$$

where the latter equality follows from the Inclusion–Exclusion principle. Finally, we have

3.4 $$\begin{aligned} |\mathscr {C}(T \setminus A)| = \frac{|\mathscr {C}|}{q^{n-|T|+|A|}}|\mathscr {C}^\perp (T^c \cup A)|, \end{aligned}$$

from which the statement of the lemma follows. In 3.4, we have used the fact that for a code $\mathscr {C}\le \mathbb {F}_q^n$ and a set $S \subseteq [n]$, we have

$$\begin{aligned} |\mathscr {C}(S)| =\frac{|\mathscr {C}|}{q^{n-|S|}}|\mathscr {C}^\perp (S^c)|. \end{aligned}$$

This follows from a more general fact about codes supported on regular lattices; see [26, Theorem 24]. For the Hamming support, an elementary proof can be obtained by considering the identity

$$\begin{aligned}(\mathscr {C}\cap \mathbb {F}_q^n(S))^\perp = \mathscr {C}^\perp + \mathbb {F}_q^n(S^c)\end{aligned}$$

and computing dimensions with the Grassmann Formula. $\square$

With the aid of Proposition 3.5 we can prove the following result, which can be seen as an analogue of a *MacWilliams binomial moment* identity.

### Proposition 3.6

Let $\mathscr {C}\le \mathbb {F}_q^n$ be of dimension *k*, $S \subseteq [n]$ and $|S| \le t \le n$. We have

$$\begin{aligned}  &   \sum _{i=0}^n \left( {\begin{array}{c}n-i\\ t-i\end{array}}\right) W_i^S(\mathscr {C}) = \\    &   q^{k-n+t-|S|}(q-1)^{|S|}\sum _{i=0}^n \sum _{D \subseteq S}\sum _{B \subseteq D} (-1)^{|D|-|B|} (1 - q)^{-|B|} \left( {\begin{array}{c}n-|S|-i+|B|\\ t-|S|\end{array}}\right) W_i^D(\mathscr {C}^{\perp }). \end{aligned}$$

### Proof

We prove the statement by first fixing a subset $S \subseteq [n]$ and summing both sides of the expression in Proposition 3.5 over all $T \subseteq [n]$ with $S \subseteq T$ and $|T|=t$. The LHS of the equality in Proposition 3.5 gives

$$\begin{aligned} \sum _{\begin{array}{c} S \subseteq T \subseteq [n] \\ |T|=t \end{array}} |\mathscr {C}(S,T)|&= \sum _{\begin{array}{c} x \in \mathscr {C}, \\ \sigma (x) \supseteq S \end{array}} |\{T \subseteq [n] : |T|=t, \, \sigma (x) \subseteq T, \, S \subseteq T\}| \\&= \sum _{i=0}^n\sum _{\begin{array}{c} x \in \mathscr {C}\\ \sigma (x) \supseteq S \\ \omega ^{\text {H}}(x) = i \end{array}} |\{T \subseteq [n] : |T|=t, \, S \subseteq \sigma (x) \subseteq T\}| \\&= \sum _{i=0}^n\sum _{\begin{array}{c} x \in \mathscr {C}\\ \sigma (x) \supseteq S \\ \omega ^{\text {H}}(x) = i \end{array}} \left( {\begin{array}{c}n-i\\ t-i\end{array}}\right) = \sum _{i=0}^n \left( {\begin{array}{c}n -i\\ t-i\end{array}}\right) W_i^S(\mathscr {C}), \end{aligned}$$

which is the LHS of the proposition. For the RHS we compute

3.5 $$\begin{aligned} \sum _{\begin{array}{c} S \subseteq T \subseteq [n]\\ |T|=t \end{array}}|\mathscr {C}| \sum _{A \subseteq S} (-1)^{|A|}\displaystyle \frac{|\mathscr {C}^\perp (T^c \cup A)|}{q^{n-t+|A|}} = |\mathscr {C}| \sum _{A \subseteq S} \displaystyle \frac{(-1)^{|A|}}{q^{n-t+|A|}} \sum _{\begin{array}{c} S \subseteq T \subseteq [n]\\ |T|=t \end{array}}|\mathscr {C}^\perp (T^c \cup A)|. \end{aligned}$$

We now write the last sum in (3.5) differently. For a fixed $A \subseteq S$ we have

$$\begin{aligned} \sum _{\begin{array}{c} S \subseteq T \subseteq [n] \\ |T|=t \end{array}}|\mathscr {C}^\perp (T^c\cup A)|&= \sum _{i=0}^n \sum _{\begin{array}{c} x \in \mathscr {C}^\perp \\ \omega ^{\text {H}}(x) = i \end{array}} |\{T \subseteq [n] : |T| =t, \sigma (x) \subseteq T^c\cup A, S \subseteq T\}| \\&= \sum _{i=0}^n \sum _{\begin{array}{c} x \in \mathscr {C}^\perp \\ \omega ^{\text {H}}(x) = i \end{array}} |\{T \subseteq [n] : |T| =n-t, \sigma (x) \subseteq T\cup A, T \subseteq S^c\}|\\&= \sum _{i=0}^n \sum _{\begin{array}{c} x \in \mathscr {C}^\perp \\ \omega ^{\text {H}}(x) = i \\ \sigma (x) \subseteq S^c \cup A \end{array}} |\{T \subseteq S^c : |T| =n-t, \sigma (x) \subseteq T\cup A\}|\\&= \sum _{i=0}^n \sum _{j=0}^{|A|} \sum _{\begin{array}{c} x \in \mathscr {C}^\perp , \\ \omega ^{\text {H}}(x) = i \\ |\sigma (x) \cap A|=j \\ |\sigma (x) \cap S^c|=i-j \end{array}} \left( {\begin{array}{c}n-|S| -i+j\\ n-t-i+j\end{array}}\right) \\&=\sum _{i=0}^n \sum _{j=0}^{|A|} \sum _{\begin{array}{c} B \subseteq A \\ |B| = j \end{array}}\, \sum _{\begin{array}{c} D \subseteq S^c, \\ |D| = i-j \end{array}}\, \sum _{\begin{array}{c} x \in \mathscr {C}^\perp \\ \sigma (x)=D \cup B \end{array}} \left( {\begin{array}{c}n-|S| -i+j\\ n-t-i+j\end{array}}\right) \\&= \sum _{i=0}^n \sum _{B \subseteq A}\sum _{\begin{array}{c} D \subseteq S^c, \\ |D| = i-|B| \end{array}} \left( {\begin{array}{c}n-|S| -|D|\\ n-t-|D|\end{array}}\right) W_i^{D \cup B}(\mathscr {C}^\perp ), \end{aligned}$$

where the last step follows from the fact that $|\{x \in \mathscr {C}^\perp : \sigma (x) = B \cup D\}| = W_i^{D \cup B}(\mathscr {C}^\perp )$ for $B \subseteq A$ with $|B|=j$ and $D \subseteq S^c$ with $|D|=i-j$. After substituting this expression into (3.5), we simplify further by reordering the summands and applying the Binomial Theorem as follows:

$$\begin{aligned}&|\mathscr {C}| \sum _{A \subseteq S} \displaystyle \frac{(-1)^{|A|}}{q^{n-t+|A|}} \sum _{i=0}^n \sum _{B \subseteq A}\sum _{\begin{array}{c} D \subseteq S^c \\ |D| = i-|B| \end{array}} \left( {\begin{array}{c}n-|S| -|D|\\ n-t-|D|\end{array}}\right) W_i^{D \cup B}(\mathscr {C}^\perp ) \\&\quad = q^{k-n+t}\sum _{i=0}^n \sum _{B \subseteq S} \sum _{\begin{array}{c} D \subseteq S^c\\ |D| = i-|B| \end{array}} \left( {\begin{array}{c}n-|S| -|D|\\ n-t-|D|\end{array}}\right) W_i^{D \cup B}(\mathscr {C}^\perp ) \sum _{B \subseteq A \subseteq S} \displaystyle \frac{(-1)^{|A|}}{q^{|A|}}\\&\quad = q^{k-n+t}\sum _{i=0}^n \sum _{B \subseteq S} \sum _{\begin{array}{c} D \subseteq S^c\\ |D| = i-|B| \end{array}} \left( {\begin{array}{c}n-|S| -|D|\\ n-t-|D|\end{array}}\right) W_i^{D \cup B}(\mathscr {C}^\perp )\sum _{j=|B|}^{|S|} (-1)^j \left( {\begin{array}{c}|S| - |B|\\ j- |B|\end{array}}\right) q^{-j}\\&\quad =q^{k-n+t}\sum _{i=0}^n \sum _{B \subseteq S} \sum _{\begin{array}{c} D \subseteq S^c\\ |D| = i-|B| \end{array}} \left( {\begin{array}{c}n-|S| -|D|\\ n-t-|D|\end{array}}\right) W_i^{D \cup B}(\mathscr {C}^\perp ) (-1)^{|S|} q^{-|B|} \\*&\qquad \cdot \sum _{j=0}^{|S|-|B|} (-1)^{|S|- |B| - j} \left( {\begin{array}{c}|S| - |B|\\ j\end{array}}\right) (q^{-1})^j\\&\quad =q^{k-n+t}\sum _{i=0}^n \sum _{B \subseteq S} \sum _{\begin{array}{c} D \subseteq S^c\\ |D| = i-|B| \end{array}} \left( {\begin{array}{c}n-|S| -|D|\\ n-t-|D|\end{array}}\right) W_i^{D \cup B}(\mathscr {C}^\perp ) (-1)^{|S|} q^{-|B|} (q^{-1}-1)^{|S|-|B|}\\&\qquad = q^{k-n+t-|S|}(q-1)^{|S|}\sum _{i=0}^n \sum _{B \subseteq S} (1 - q)^{-|B|} \sum _{\begin{array}{c} D \subseteq S^c\\ |D| = i-|B| \end{array}} \left( {\begin{array}{c}n-|S| -i +|B|\\ t-|S|\end{array}}\right) W_i^{D \cup B}(\mathscr {C}^{\perp }). \end{aligned}$$

To conclude the proof we will need the following claim.

### Claim A

Let $\mathscr {C}\le \mathbb {F}_q^n$, $S \subseteq [n]$ with $|S| = t$, $B \subseteq S$, and $1 \le j \le n$. Then

$$\begin{aligned}\sum _{\begin{array}{c} D \subseteq S^c \\ |D| = j- |B| \end{array}} W_j^{D \cup B}(\mathscr {C}^{\perp }) = \sum _{B \subseteq D \subseteq S}(-1)^{|D| - |B|} W_j^D(\mathscr {C}^{\perp }).\end{aligned}$$

### Proof of the claim

For all $D \subseteq S^c$ with $|D| = j- |B|$ we have

$$\begin{aligned}W_j^{D \cup B}(\mathscr {C}^{\perp }) = \left| \{v \in \mathscr {C}^{\perp } \,: \, \sigma (v) = D \cup B\} \right| .\end{aligned}$$

Hence

$$\begin{aligned} \sum _{\begin{array}{c} D \subseteq S^c \\ |D| = j-|B| \end{array}}W_j^{D \cup B}(\mathscr {C}^{\perp })&= \left| \{v \in \mathscr {C}^{\perp } \, : \, D \subseteq S^c, \, |D| = j-|B|, \, \sigma (v) = D \cup B \} \right| \\&= \left| \{v \in \mathscr {C}^{\perp } \, : \, B \subseteq D \subseteq [n]\setminus (S \setminus B), \, |D| = j, \, \sigma (v) = D \} \right| \\&= \left| \{ v \in \mathscr {C}^{\perp } \, : \, B \subseteq D \subseteq [n], \, |D|=j, \, \sigma (v) = D\} \right| - \\&\left| \bigcup _{\emptyset \subsetneq A \subseteq (S\setminus B)} \{v \in \mathscr {C}^{\perp } \, : \, B\cup A \subseteq D \subseteq [n], \, |D|=j, \, \sigma (v) = D\} \right| . \end{aligned}$$

Using the Inclusion–Exclusion principle followed by Lemma 3.3, we get

$$\begin{aligned}\sum _{\begin{array}{c} D \subseteq S^c \\ |D| = j-|B| \end{array}}W_j^{D \cup B}(\mathscr {C}^{\perp })&= \sum _{\emptyset \subseteq A \subseteq S\setminus B}(-1)^{|A|} \sum _{\begin{array}{c} A \cup B \subseteq D \subseteq [n]\\ |D| = j \end{array}} W_j^D(\mathscr {C}^{\perp })\\&= \sum _{\emptyset \subseteq A \subseteq S \setminus B}(-1)^{|A|} W_j^{A \cup B}(\mathscr {C}^{\perp })\\&= \sum _{B \subseteq D \subseteq S}(-1)^{|D|-|B|}W_j^D(\mathscr {C}^{\perp }), \end{aligned}$$

proving the desired claim.

Finally, from Claim A it follows that

$$\begin{aligned} q^{k-n+t-|S|}(q-1)^{|S|}\sum _{i=0}^n \sum _{B \subseteq S} (1 - q)^{-|B|} \sum _{\begin{array}{c} D \subseteq S^c\\ |D| = i-|B| \end{array}} \left( {\begin{array}{c}n-|S| -i +|B|\\ t-|S|\end{array}}\right) W_i^{D \cup B}(\mathscr {C}^{\perp }) \\= q^{k-n+t-|S|}(q-1)^{|S|}\sum _{i=0}^n \sum _{D \subseteq S}\sum _{B \subseteq D} (-1)^{|D|-|B|} (1 - q)^{-|B|} \left( {\begin{array}{c}n-|S|-i+|B|\\ t-|S|\end{array}}\right) W_i^D(\mathscr {C}^{\perp }), \end{aligned}$$

concluding the proof. $\square$

In order to “isolate” the refined weight distribution of a code $\mathscr {C}\le \mathbb {F}_q^n$ in the sum of Proposition 3.6, we will use the following lemma. This is the well-known *q*-Binomial Inversion Formula and can be found for example in [2].

### Lemma 3.7

Let

$$\begin{aligned}\alpha _t:=\sum _{i=0}^n \left( {\begin{array}{c}n-i\\ t-i\end{array}}\right) \beta _i \quad \text {for}\, 0 \le t \le n.\end{aligned}$$

Then

$$\begin{aligned}\beta _i = \displaystyle \sum _{t=0}^n (-1)^{i-t} \left( {\begin{array}{c}n-t\\ i-t\end{array}}\right) \alpha _t \quad \text {for all}\, 0 \le i \le n. \end{aligned}$$

We can finally establish the MacWilliams-type identity for the refined weight distribution of a code.

### Theorem 3.8

Let $\mathscr {C}\le \mathbb {F}_q^n$ be of dimension *k* and fix $S \subseteq [n]$. For all $0 \le i \le n$ we have

3.6 $$\begin{aligned} \begin{array}{c} W_i^S(\mathscr {C}) = q^{k-n - |S|} (q-1)^{|S|} \sum _{t=|S|}^n \sum _{j=0}^n \sum _{D \subseteq S} \sum _{B \subseteq D} (-1)^{i-t+|D|} q^t\\ (q-1)^{-|B|}\left( {\begin{array}{c}n-t\\ i-t\end{array}}\right) \left( {\begin{array}{c}n-|S|-j+|B|\\ t - |S|\end{array}}\right) W_j^D(\mathscr {C}^{\perp }).\end{array}\end{aligned}$$

### Proof

The statement follows directly from Proposition 3.6 and Lemma 3.7, by setting $\beta _i = W_i^S(\mathscr {C})$ and

$$\begin{aligned}\alpha _t = q^{k-n+t-|S|}(q-1)^{|S|}\sum _{j=0}^n \sum _{D \subseteq S}\sum _{B \subseteq D} (-1)^{|D|-|B|} (1 - q)^{-|B|} \left( {\begin{array}{c}n\!-\!|S|\!-\!j\!+\!|B|\\ t-|S|\end{array}}\right) W_j^D(\mathscr {C}^{\perp }). \end{aligned}$$

$$\square $$

### Remark 3.9

Although we call the above a MacWilliams-type identity, enumerators we consider do not naturally fit in the framework of the MacWilliams identities, as they do not represent the cardinalities of the blocks of a partition of the underlying code; see [11]. However, one can see the above as a generalization of the classical MacWilliams identity. In fact, with tedious but straightforward computations one can show that (3.6) reduces to the famous MacWilliams identity [23] when $S = \emptyset$.

When applying MacWilliams-type identities, it is crucial to understand the dependencies between variables and which groups of these determine each other, possibly under extra assumptions. The next result shows that $W_i^S(\mathscr {C})$ can be rewritten in terms of $W_j^T(\mathscr {C}^{\perp })$ for $1 \le j \le n$ and all $T \subseteq [n]$ with $|T| = |S|$, provided that the cardinality of *S* does not exceed the minimum distance of $\mathscr {C}^\perp$. This result will become especially useful for the linear programming bounds presented in Sect. 4.

### Corollary 3.10

Let $\mathscr {C}\le \mathbb {F}_q^n$ be a code of dimension *k*, $d^{\perp }$ the minimum distance of $\mathscr {C}^{\perp }$, and $S \subseteq [n]$. If $|S| \le d^{\perp }$, then

$$\begin{aligned} W_i^S(\mathscr {C})&= \left( {\begin{array}{c}n-|S|\\ i-|S|\end{array}}\right) q^{k-n}(q-1)^{i} + q^{k-n-|S|}(q-1)^{|S|}\sum _{t=|S|}^n \sum _{j=d^{\perp }}^n \sum _{\begin{array}{c} T \subseteq [n]\\ |T|=|S| \end{array}}\\&\quad \times \sum _{D \subseteq S\cap T}\sum _{B \subseteq D}(-1)^{i-t+|D|}\\  &\quad q^t(q-1)^{-|B|} \left( {\begin{array}{c}n-t\\ i-t\end{array}}\right) \left( {\begin{array}{c}n-|S|-j+|B|\\ t-|S|\end{array}}\right) \left( {\begin{array}{c}j-|D|\\ |S|-|D|\end{array}}\right) ^{-1}W_j^T(\mathscr {C}^{\perp }). \end{aligned}$$

### Proof

Let $s:= |S|$ and consider the identity from Theorem 3.8. We first isolate the case $j=0$. Note that $W_0^D(\mathscr {C}^{\perp }) =1$ if $D = \emptyset$ and $W_0^D(\mathscr {C}^{\perp }) = 0$ otherwise. Hence the summand of Theorem 3.8 corresponding to $j=0$ is:

$$\begin{aligned} \sum _{t = s}^n (-1)^{i-t}q^t\left( {\begin{array}{c}n-t\\ i-t\end{array}}\right) \left( {\begin{array}{c}n-s\\ n-t\end{array}}\right) =&\sum _{t = s}^n (-1)^{i-t}q^t\left( {\begin{array}{c}n-s\\ n-i\end{array}}\right) \left( {\begin{array}{c}i-s\\ i-t\end{array}}\right) \\ =&\left( {\begin{array}{c}n-s\\ n-i\end{array}}\right) \sum _{t=s}^{i}(-1)^{i-t}q^t\left( {\begin{array}{c}i-s\\ i-t\end{array}}\right) \\ =&\left( {\begin{array}{c}n-s\\ n-i\end{array}}\right) q^s \sum _{t=0}^{i-s}(-1)^{i-s-t}q^t\left( {\begin{array}{c}i-s\\ t\end{array}}\right) \\ =&\left( {\begin{array}{c}n-s\\ n-i\end{array}}\right) q^s (q-1)^{i-s}, \end{aligned}$$

where the former equality follows from the identity

$$\begin{aligned}\left( {\begin{array}{c}a\\ b+c\end{array}}\right) \left( {\begin{array}{c}b+c\\ b\end{array}}\right) = \left( {\begin{array}{c}a\\ c\end{array}}\right) \left( {\begin{array}{c}a-c\\ b\end{array}}\right) ,\end{aligned}$$

and the latter one follows from the Binomial Theorem.

Now assume $j \ge 1$. If $j \le d^{\perp }$ then $W_j^D(\mathscr {C}^{\perp }) = 0$. Thus it suffices to consider the summands for $d^{\perp } \le j \le n$. By assumption $s \le d^{\perp }$. Hence for all $D \subseteq S$ we can apply Lemma 3.3 and get

$$\begin{aligned}W_j^D(\mathscr {C}^{\perp }) = \left( {\begin{array}{c}j-|D|\\ s-|D|\end{array}}\right) ^{-1}\sum _{\begin{array}{c} D \subseteq T \subseteq [n]\\ |T|=s \end{array}}W_j^T(\mathscr {C}^{\perp }).\end{aligned}$$

Therefore for $j \ge d^{\perp }$, (3.6) can be rewritten as

$$\begin{aligned}&\sum _{j=d^{\perp }}^n \sum _{t=s}^n \sum _{D \subseteq S} \sum _{B \subseteq D} (-1)^{i-t+|D|}q^t(q-1)^{-|B|}\left( {\begin{array}{c}n-t\\ i-t\end{array}}\right) \left( {\begin{array}{c}n-s-j+|B|\\ t-s\end{array}}\right) \left( {\begin{array}{c}j-|D|\\ s-|D|\end{array}}\right) ^{-1}\\&\qquad \times \sum _{\begin{array}{c} D \subseteq T \subseteq [n]\\ |T|=s \end{array}}W_j^T(\mathscr {C}^{\perp })\\&\quad =\sum _{j=d^{\perp }}^n \sum _{t=s}^n \sum _{\begin{array}{c} T \subseteq [n]\\ |T|=s \end{array}} \sum _{D \subseteq S\cap T}\sum _{B \subseteq D}(-1)^{i-t+|D|}q^t(q-1)^{-|B|}\left( {\begin{array}{c}n-t\\ i-t\end{array}}\right) \left( {\begin{array}{c}n-s-j+|B|\\ t-s\end{array}}\right) \\  &\qquad \times \left( {\begin{array}{c}j-|D|\\ s-|D|\end{array}}\right) ^{-1}W_j^T(\mathscr {C}^{\perp }). \end{aligned}$$

Putting everything together proves the desired statement. $\square$

Note that in the above proof the assumption $|S| \le d^{\perp }$ was needed to apply Lemma 3.3. A natural question is whether $W_i^S(\mathscr {C})$ can be fully expressed in terms of $W_j^T(\mathscr {C}^{\perp })$, for $0 \le j \le n$ and $|T| = |S|$ when $|S| > d^{\perp }$. At the time of writing this paper, we are unable to answer this question.

## Applications and bounds

Delsarte’s linear programming (LP) bound [8] is a powerful tool to estimate the size of a code with a certain length and minimum distance. It combines the classical MacWilliams identities with, as the name suggests, linear programming. In this section, we study a new LP bound for codes with locality. We use our main duality result, Theorem 3.8, to build a linear program that gives a bound on the size of $(r,\delta )$-LRCs.

### Notation 4.1

In the sequel, for a code $\mathscr {C}\le \mathbb {F}_q^n$, $0 \le i \le n$ and $1 \le j \le n$, we will write $\smash {W_i^j(\mathscr {C})}$ instead of $\smash {W_i^{\{j\}}(\mathscr {C})}$.

The next result shows that it is possible to bound from below, in any $(r, \delta )$-LRC, the number of codewords with coordinate *j* in their support and weight at most $r+\delta -1$.

### Proposition 4.2

Let $\delta \ge 2$ and let $\mathscr {C}\le \mathbb {F}_q^n$ be an $(r, \delta )$-LRC, with dimension *k*. Then for all $j \in [n]$ we have

$$\begin{aligned}\sum _{i=0}^{r+\delta -1} W^j_i(\mathscr {C}^{\perp }) \ge q^{\delta -1} - q^{\delta -2}.\end{aligned}$$

### Proof

Let $j \in [n]$ and $S_j \subseteq [n]$ be minimal with respect to inclusion such that $|S_j| \le r +\delta -1$, $j \in S_j$, and $d(\pi _{S_j}(\mathscr {C})) \ge \delta$. Let $k_j:= \dim (\pi _{S_j}(\mathscr {C}))$, $d_j:= d(\pi _{S_j}(\mathscr {C}))$, and $k_j^{\perp }:= |S_j| - k_j$. From the Singleton Bound we have $|S_j|-k_j+1\ge \delta$, which means $k_j^\perp \ge \delta -1$. Furthermore there exists $v \in \mathscr {C}^{\perp }(S_j)$ such that $j \in \sigma (v)$, since otherwise we arrive to the contradiction that for all $v \in \mathscr {C}^{\perp }(S_j)$ we have $v_j=0$ and $d(\pi _{S_j}(\mathscr {C})) =1 < \delta$. These two facts together imply that there are at least $q^{\delta -1} - q^{\delta - 2}$ codewords in $\mathscr {C}^{\perp }(S_j)$ containing *j* in their support and of weight at most $|S_j| \le r+ \delta -1$. Therefore $\sum _{i=0}^{r+\delta -1} W^j_i(\mathscr {C}^{\perp }) \ge q^{\delta -1} - q^{\delta - 2}$. $\square$

Unfortunately, the converse of this statement is not true, unless $\delta =2$ (leading to the statement of Lemma 4.4 below). Consider the following example.

### Example 4.3

Let *G* and *H* be matrices over $\mathbb {F}_2$ defined as follows:

$$\begin{aligned}G:= \begin{pmatrix}1 &  1 &  0 &  1&  0 \\ 0 &  1 &  1 &  0 & 0 \\ 0 & 0 &  0& 1 &  1 \end{pmatrix}, \quad H:= \begin{pmatrix} 1 &  1 &  1 &  0 &  0 \\ 1 &  0 & 0 &  1 &  1 \end{pmatrix}.\end{aligned}$$

Let $\mathscr {C}=\{xG: x \in \mathbb {F}_2^3\}$ which then implies $\mathscr {C}^\perp =\{xH: x \in \mathbb {F}_2^2\}$. If we fix $r:=2$ and $\delta :=3$, then $r + \delta -1 = 4$ and $q^{\delta -1} - q^{\delta -2} =2$. One can easily check that for all $j \in [5]$ we have

$$\begin{aligned}\sum _{i=0}^{r+ \delta -1}W_i^j(\mathscr {C}^{\perp }) =\sum _{i=0}^{4}W_i^j(\mathscr {C}^{\perp }) = 2.\end{aligned}$$

However, for the coordinate $\{1\}$ there exists no set $S_1 \subseteq [5]$ such that $1 \in S_1$, $|S_1| \le 4$, and $d(\pi _{S_1}(\mathscr {C})) \ge 3$. In fact any projection of $\mathscr {C}$ onto a set of coordinate of size less or equal to 4 will have minimum distance 1 or 2. Hence the code $\mathscr {C}$ is not an (2, 3)-LRC.

As already mentioned, for $\delta =2$ the converse of Proposition 4.2 is also true. As a byproduct, the result also gives a characterization of codes with locality.

### Lemma 4.4

A non-degenerate code $\mathscr {C}\le \mathbb {F}_q^n$ has locality *r* if and only if $\sum _{i=2}^{r+1}W_{i}^j(\mathscr {C}^\perp ) \ge q-1$ for all $1 \le j \le n$.

Proposition 4.2 and Lemma 4.4 indicate that the MacWilliams-type identity of Corollary 3.10 becomes especially useful in the context of LRCs when considering the weight distribution $W_i^{j}(\mathscr {C}^{\perp })$, where $1 \le j \le n$ and $0 \le i \le n$. By applying Corollary 3.10, we get the following corollary.

### Corollary 4.5

Let $\mathscr {C}\le \mathbb {F}_q^n$ be a code and $l \in [n]$. Then

$$\begin{aligned}\begin{array}{c}W_i^l(\mathscr {C}) = \left( {\begin{array}{c}n-1\\ i-1\end{array}}\right) q^{k-n}(q-1)^{i} + q^{k-n-1}(q-1) \sum _{j=d^{\perp }}^n \sum _{s=1}^n \sum _{t=1}^i (-1)^{i-t}q^t\left( {\begin{array}{c}n-t\\ i-t\end{array}}\right) \\ \left( \frac{1}{j}\left( {\begin{array}{c}n-1-j\\ t-1\end{array}}\right) \right) ^{1-\delta (l,s)} \left( \frac{1-j}{j}\left( {\begin{array}{c}n-1-j\\ t-1\end{array}}\right) - (q-1)^{-1}\left( {\begin{array}{c}n-j\\ t-1\end{array}}\right) \right) ^{\delta (l,s)}W_j^s(\mathscr {C}^{\perp }), \end{array}\end{aligned}$$

where $\delta (l,s) =1$ if $l=s$ and 0 otherwise.

### Proof

Using Corollary 3.10 for $S = \{l\}$ we immediately get

$$\begin{aligned}\begin{array}{c}W_i^{l}(\mathscr {C}) = \left( {\begin{array}{c}n-1\\ i-1\end{array}}\right) q^{k-n}(q-1)^i + q^{k-n-1}(q-1)\sum _{t=1}^n \sum _{j=d^{\perp }}^n \sum _{\begin{array}{c} T \subseteq [n]\\ |T|=1 \end{array}} \sum _{D \subseteq \{l\}\cap T}\sum _{B \subseteq D}(-1)^{i-t+|D|}q^t\\ (q-1)^{-|B|}\left( {\begin{array}{c}n-t\\ i-t\end{array}}\right) \left( {\begin{array}{c}n-1-j+|B|\\ t-1\end{array}}\right) \left( {\begin{array}{c}j-|D|\\ 1-|D|\end{array}}\right) ^{-1}W_j^T(\mathscr {C}^{\perp }). \end{array}\end{aligned}$$

We now compute

4.1 $$\begin{aligned} \sum _{D \subseteq \{l\}\cap T}\sum _{B \subseteq D}(-1)^{|D|}(q-1)^{-|B|}\left( {\begin{array}{c}n-1-j+|B|\\ t-1\end{array}}\right) \left( {\begin{array}{c}j-|D|\\ 1-|D|\end{array}}\right) ^{-1}, \end{aligned}$$

for both $T = \{l\}$ and $T = \{s\}$ where $s \ne l$. In the former case, (4.1) is equal to

$$\begin{aligned}&\frac{1}{j}\left( {\begin{array}{c}n-1-j\\ t-1\end{array}}\right) - \left[ (q-1)^{-1}\left( {\begin{array}{c}n-j\\ t-1\end{array}}\right) + \left( {\begin{array}{c}n-1-j\\ t-1\end{array}}\right) \right] . \end{aligned}$$

In the case $T= \{s\}$ where $s \ne l$, (4.1) is equal to $$\frac{1}{j}\left( {\begin{array}{c}n-1-j\\ t-1\end{array}}\right) $$. The results then follows. $\square$

### Remark 4.6

There exist other methods to derive the MacWilliams-type identity of Corollary 4.5. A first approach was established in [14], where the parameter $W_i^l(\mathscr {C})$ is rewritten in terms of the $W_j(\mathscr {C})$’s and $W_j(\mathscr {C}([n]\setminus \{l\}))$’s for $1 \le j, \, l \le n$. The classical MacWilliams identity is then applied to the latter terms. In addition, it was brought to our attention by M. Grassl that our refined weight distribution seems related to the notion of *split weight enumerator*; see [29] for more details. We were able to show that this is in fact the case and are able to derive Corollary 4.5 also as a corollary of [29, Proposition 1].

The last result we need to apply a linear program is the following, which is a specific case of Lemma 3.3 for $A = \emptyset$ and $|S| = 1$.

### Lemma 4.7

Let $\mathscr {C}\le \mathbb {F}_q^n$ be non-degenerate. For all $1 \le i \le n$ and $1\le j \le n$ we have

$$\begin{aligned}W_i(\mathscr {C}) = {\displaystyle \sum _{j=1}^n W_i^j (\mathscr {C})}/{i}.\end{aligned}$$

For ease of exposition, we introduce the following notation.

### Notation 4.8

Let $A=\{a_{il} \mid 1\le i \le n+1, \, 1\le l \le n\} \subseteq \mathbb {R}_{\ge 0}$. For $1 \le i,l \le n$ we denote by $a_{il}^\perp$ the following linear combination of elements of *A*:

$$\begin{aligned}\begin{array}{c} a_{il}^\perp = \left( {\begin{array}{c}n-1\\ i-1\end{array}}\right) (q-1)^{i} + q^{-1}(q-1) \sum _{j=d^{\perp }}^n \sum _{s=1}^n \sum _{t=1}^i (-1)^{i-t}q^t\left( {\begin{array}{c}n-t\\ i-t\end{array}}\right) \cdot \\ \left( \frac{1}{j}\left( {\begin{array}{c}n-1-j\\ t-1\end{array}}\right) \right) ^{1-\delta (l,s)} \left( \frac{1-j}{j}\left( {\begin{array}{c}n-1-j\\ t-1\end{array}}\right) - (q-1)^{-1}\left( {\begin{array}{c}n-j\\ t-1\end{array}}\right) \right) ^{\delta (l,s)}a_{js}. \end{array}\end{aligned}$$

The following result gives a linear program that establishes bounds for codes with $(r,\delta )$-locality.

### Theorem 4.9

(LP bound for $(r,\delta )$-LRCs) Let $\mathscr {C}\le \mathbb {F}_q^n$ be a non-degenerate code of minimum distance at least *d*, dimension *k*, and $(r,\delta )$-locality. Let $\mu ^*$ denote the minimum value of

$$\begin{aligned}\sum _{i=1}^{n}\left( {\displaystyle \sum _{j=1}^{n} a_{ij}}/{i}\right) ,\end{aligned}$$

where $a_{ij} \in \mathbb {R}$, for $1 \le i \le n+1$ and $1 \le j \le n$, satisfy the following constraints:

(i) $$a_{ij}\ge 0$$ for $$1 \le i,j \le n$$,
(ii) $$a_{ij}^\perp \ge 0$$ for $$1 \le i,j \le n$$,
(iii) $a_{ij}^\perp =0$ for $1 \le i \le d-1$ and $1 \le j \le n$,
(iv) $$\smash {\textstyle \sum _{i=1}^{r+\delta -1}a_{ij} \ge q^{\delta -1}-q^{\delta -2}}$$ for $$1 \le j \le n$$,
(v) $$a_{1j} =0$$ for $$1 \le j \le n$$.

Then

$$\begin{aligned}k \le n- \lceil \log _q(1+\mu ^*) \rceil .\end{aligned}$$

### Proof

We claim that for any non-degenerate linear code $\mathscr {C}\le \mathbb {F}_q^n$ of minimum distance *d* and $(r,\delta )$-locality, the assignment $\smash {a_{ij} = W_i^j(\mathscr {C}^\perp )}$ is a feasible solution of the linear program. Indeed, (i) and (ii) are satisfied trivially. Constraint (iii) guarantees that the minimum distance of the code is at least *d* and constraint (iv) needs to be fulfilled in order for the code to be an $(r,\delta )$-LRC by Proposition 4.2. Finally constraint (v) makes sure that $\mathscr {C}$ is non-degenerate. Therefore the optimum of the linear program gives a lower bound on the size of $\mathscr {C}^\perp$ by Lemma 4.7. $\square$

We conclude this section with some results obtained with the LP bound of Theorem 4.9 in Tables 1, 2, 3, 4 and 5. For fixed parameters $(q,n,d,r,\delta )$, we give an upper bound on the dimension *k* of an $(r,\delta )$-LRC in $\mathbb {F}_q^n$ and minimum distance at least *d*. The computations were performed using SageMath. In the case where $\delta =2$ (i.e. Tables 1, 2, 3 and 4) we compare our results to (2.1) (**gen. Singl.**) and (2.2) (**SH**). The latter is computed in two different ways: first, the value $\smash {k_{\text {opt}}^{(q)}}$ is estimated using a classical LP bound (**SH with LP**); second, the exact value of $\smash {k_{\text {opt}}^{(q)}}$ is computed (**SH exact**), since it can be determined for the parameters considered in the tables by looking at databases of codes. The most fair comparison between our result and (2.2) is given by SH with LP, since the value of SH exact is only known for very small parameters. Our results show that Theorem 4.9 is often tighter than (2.2) (this is indicated in red on the tables). A similar comparison is done in Table 5, but using (2.3) and (2.4) instead.

### Remark 4.10

In [19], the authors establish a different LP-type bound, which applies to codes with recovery sets that are disjoint and of equal size, requiring, in particular, $r+\delta -1$ to divide *n*; see [19, Section IV]. Since the LP bound presented in this paper does not require those properties, it is technically not comparable with the one of [19]. Note moreover that the requirement about the disjoint recovery sets is not captured by the fundamental code parameters $(q,n,d,r,\delta )$, which are the only input needed for our LP bound.

In fact, the assumptions required in [19] reduce the set of parameters for which the bound can be applied. For example, in the case where $n=6$, $r=2$, $d=3$, and $q=2$, Table 1 of [19] states that the largest dimension of an LRC code with disjoint recovery sets is 2. However, the code

$$\begin{aligned}\mathscr {C}:= \left\langle (1, 1, 0, 0,0,1), \, (0, 0, 0, 1, 1,1), \, (1, 0, 1, 1,0,0)\right\rangle \end{aligned}$$

has parameter set $n=6$, $r=2$, $d=3$, $q=2$, but has dimension 3. Note that this does not contradict [19], since $\mathscr {C}$ does not have disjoint recovery sets.

**Table 1:** Caption: $$q=2$$, $$\delta =2$$

*n*	*d*	*r*	LP	SH with LP	SH exact	gen. Singl
10	4	2	$k \le {\textbf {4}}$	$k \le 5$	$k \le 5$	$k \le 5$
11	3	3	$k \le {\textbf {6}}$	$k \le 7$	$k \le 7$	$k \le 7$
12	3	3	$k \le {\textbf {7}}$	$k \le 8$	$k \le 8$	$k \le 8$
14	4	6	$k \le {\textbf {8}}$	$k \le 9$	$k \le 9$	$k \le 10$
17	7	2	$k \le {\textbf {5}}$	$k \le 6$	$k \le 6$	$k \le 8$
18	8	2	$k\le {\textbf {5}}$	$k \le 6$	$k \le 6$	$k \le 8$
20	9	5	$k \le 5$	$k \le 6$	$k \le 5$	$k \le 11$
20	11	2	$k \le {\textbf {2}}$	$k \le 3$	$k \le 3$	$k \le 7$

We present an explicit code that meets our LP bound with equality for a parameter regime presented in Table 1. This shows that the bound is sharp in this case and potentially in others.

### Example 4.11

Let $\mathscr {C}\le \mathbb {F}_2^{10}$ be the code with the following generator matrix.

$$\begin{aligned}G:= \begin{pmatrix} 1 &  0 & 1 &  1 &  1 &  0 & 0 & 0 & 0 & 0 \\ 1 &  1 & 0 & 1& 0 & 1 & 0 & 0 & 0 & 0 \\ 0& 1 &  1 &  0 &  0 & 0 & 0& 1& 1& 0 \\ 1& 0& 1& 0& 0& 0& 1& 1& 0& 1 \end{pmatrix}. \end{aligned}$$

One can check that this codes has dimension $k=4$, minimum distance $d=4$, and locality $r=2$. This shows that our LP bound is tight for some parameters.

**Table 2:** q=3, $$\delta =2$$

*n*	*d*	*r*	LP	SH with LP	SH exact	gen. Singl
10	2	4	$k \le {\textbf {7}}$	$k \le 8$	$k \le 8$	$k \le 8$
11	6	4	$k \le {\textbf {4}}$	$k \le 5$	$k \le 5$	$k \le 5$
13	9	2	$k \le {\textbf {2}}$	$k \le 3$	$k \le 3$	$k \le 4$
14	2	6	$k \le {\textbf {11}}$	$k \le 12$	$k \le 12$	$k \le 12$
14	9	3	$k \le 3$	$k \le 4$	$k \le 3$	$k \le 5$
18	6	5	$k \le 10$	$k \le 11$	$k \le 10$	$k \le 11$
25	5	5	$k \le {\textbf {15}}$	$k \le 17$	$k \le 17$	$k \le 18$

**Table 3:** Caption: $$q=4$$, $$\delta =2$$

*n*	*d*	*r*	LP	SH with LP	SH exact	gen. Singl
9	3	3	$k \le {\textbf {5}}$	$k \le 6$	$k \le 6$	$k \le 6$
10	2	4	$k \le {\textbf {7}}$	$k \le 8$	$k \le 8$	$k \le 8$
11	8	2	$k \le 2$	$k \le 3$	$k \le 2$	$k \le 3$
12	2	5	$k \le {\textbf {9}}$	$k \le 10$	$k \le 10$	$k \le 10$
14	2	6	$k \le {\textbf {11}}$	$k \le 12$	$k \le 12$	$k \le 12$
15	10	4	$k \le 4$	$k \le 5$	$k \le 4$	$k \le 5$
15	8	6	$k \le 6$	$k \le 7$	$k \le 6$	$k \le 7$
20	2	9	$k \le {\textbf {17}}$	$k \le 18$	$k \le 18$	$k \le 18$

**Table 4:** $${q=5}$$, $${\delta =2}$$

*n*	*d*	*r*	LP	SH with LP	SH exact	gen. Singl
9	3	3	$k \le {\textbf {5}}$	$k \le 6$	$k \le 6$	$k \le 6$
11	3	4	$k \le {\textbf {7}}$	$k \le 8$	$k \le 8$	$k \le 8$
14	2	6	$k \le {\textbf {11}}$	$k \le 12$	$k\le 12$	$k \le 12$
16	2	7	$k \le {\textbf {13}}$	$k \le 14$	$k \le 14$	$k \le 14$
18	2	8	$k \le {\textbf {15}}$	$k \le 16$	$k \le 16$	$k \le 16$
22	2	10	$k \le {\textbf {19}}$	$k \le 20$	$k \le 20$	$k \le 20$
24	2	11	$k \le {\textbf {21}}$	$k \le 22$	$k \le 22$	$k \le 22$

**Table 5:** $$q=2$$, $$\delta =3$$

*n*	*d*	*r*	LP	SH with LP	SH exact	gen. Singl
15	7	5	$k \le 5$	$k \le 5$	$k \le 5$	$k \le 7$
16	5	5	$k \le 8$	$k \le 7$	$k \le 7$	$k \le 10$
16	8	5	$k \le {\textbf {4}}$	$k \le 5$	$k \le 5$	$k \le 7$
17	9	5	$k \le 3$	$k \le 3$	$k \le 3$	$k \le 7$
17	10	5	$k \le 2$	$k \le 2$	$k \le 2$	$k \le 6$
18	7	5	$k \le 7$	$k \le 7$	$k \le 7$	$k \le 10$
19	8	5	$k \le 7$	$k \le 7$	$k \le 7$	$k \le 10$
20	5	7	$k \le 13$	$k \le 11$	$k \le 11$	$k \le 14$

### Remark 4.12

As Table 5 illustrates, when $\delta > 2$ our LP bound does not seem to beat the shortening bound (both the exact and the LP one) as frequently as for $\delta =2$. A possible explanation for why this happens is that, for $\delta > 2$, the $(r,\delta )$-LRCs are not fully characterized by the constraint (iv) in Theorem 4.9. Hence a code that achieves the minimum of the objective function may not be the dual code of an $(r, \delta )$-LRC.

## The role of the field size

In this section we establish various bounds for LRCs that involve the underlying field size *q*. The goal is to understand the role played by this parameter for the code’s locality.

We first use the refined weight distribution introduced in Sect. 3 to derive bounds connecting the dual distance with the locality and the field size. We then apply the theory of generalized weights to derive bounds involving the length, dimension, minimum distance, locality, and field size.

To derive our first bound for LRCs, recall from Definition 3.4 that

$$\begin{aligned}\smash {\mathscr {C}(S, [n]) = \{x \in \mathscr {C}: S \subseteq \sigma (x)\}}.\end{aligned}$$

We will need the following lemma.

### Lemma 5.1

Let $\mathscr {C}\le \mathbb {F}_q^n$ be a code of dimension *k* and let $S \subseteq [n]$ with $|S|:=s \le k$. There exists $x \in \mathscr {C}(S,[n])$ with $\omega ^{\text {H}}(x) \le n-k+s$.

### Proof

Without loss of generality we may assume $S =\{1,\dots ,s\}$. Consider the generator $G \in \mathbb {F}_q^{k \times n}$ of $\mathscr {C}$ to be in systematic form and let $g_1,\dots , g_k \in \mathbb {F}_q^n$ denote the rows of *G*. Then $x=\sum _{i=1}^sg_i$ is in $\mathscr {C}(S,[n])$ and

$$\begin{aligned}\omega ^{\text {H}}(x)=\omega ^{\text {H}}(\pi _{[k]}(x)) + \omega ^{\text {H}}(\pi _{[n]\setminus [k]}(x)) = s + \omega ^{\text {H}}(\pi _{[n]\setminus [k]}(x)) \le s+n-k,\end{aligned}$$

which proves the statement of the lemma. $\square$

The following result holds for any linear code and does not depend on the locality parameter.

### Proposition 5.2

Let $\mathscr {C}\le \mathbb {F}_q^n$ be a code of dimension *k* and minimum distance *d*. Let $S \subseteq [n]$ with $|S|\le k-1$. If $|\mathscr {C}(S,[n])| > 0$, then there exists $x \in \mathscr {C}(S,[n])$ such that

5.1 $$\begin{aligned} \omega ^{\textrm{H}}(x) \le n - k +|S| - (d-q)/q. \end{aligned}$$

### Proof

Let $s=|S|$ and $x \in \mathscr {C}(S,[n])$ of minimum weight with $x \ne 0$.

Let $t= \omega ^{\text {H}}(x)$. Without loss of generality, we may assume $S= \{1, \ldots , s\}$, $T= \{1, \ldots , t\}$, and $x = \sum _{i=1}^t e_i$, where $e_i$ is the *i*-th standard basis element. By Lemma 5.1 we have $t \le n - k + s$. Furthermore, since $\dim (\mathscr {C}) = k$, for any $A \subseteq [n]$ with $|A| \le k-1$ there exists a codeword $y \in \mathscr {C}$ such that $\sigma (y) \subseteq [n] \setminus A$. Let $A:= S \cup \{n-k+s+2, \ldots , n\}$ and note that $|A| \le k-1$. Thus, we can fix $y \in \mathscr {C}$ such that $\sigma (y) \subseteq [n]\setminus A$. Furthermore, we have

$$\begin{aligned} \omega ^{\text {H}}(\pi _T(y)) + (n-t-k+s+1) \ge \omega ^{\text {H}}(\pi _T(y)) +\omega ^{\text {H}}(\pi _{[n]\setminus T}(y)) \ge \omega ^{\text {H}}(y) \ge d, \end{aligned}$$

which gives

5.2 $$\begin{aligned} \omega ^{\text {H}}(\pi _T(y)) \ge d- (n-t) + k-s-1.\end{aligned}$$

By the pigeonhole principle, there exists $\alpha \in \mathbb {F}_q^*$ such that

$$\begin{aligned}|\{j \,: \, \pi _T(y)_j = \alpha \}| \ge (d-n+t+k -s-1)/(q-1).\end{aligned}$$

Let $x' = \alpha x - y$. Then $x'_i = \alpha x_i-y_i \ne 0$ for all $i \in S$, since $x_i \ne 0$ and $y_i = 0$. Hence

$$\begin{aligned} \omega ^{\text {H}}(x')&= \omega ^{\text {H}}(\pi _T(x')) + \omega ^{\text {H}}(\pi _{[n] \setminus T}(x'))\\&\le t - \frac{d-n+t+k-s-1}{q-1} + (n-t) - (k-s-1)\\&= n-k+s+1 - \frac{d-n+t+k-s-1}{q-1}, \end{aligned}$$

where the bound on $\omega ^{\text {H}}(\pi _T(x'))$ follows from (5.2), and the bound on $\omega ^{\text {H}}(\pi _{[n] \setminus T}(x'))$ follows from the fact that the last $k-s-1$ coordinates of both *x* and *y* are 0. Since $S \subseteq \sigma (x')$, by choice of *x* we have $\omega ^{\text {H}}(x) = t \le \omega ^{\text {H}}(x')$, which implies that

$$\begin{aligned} t \le n-k+s+1 - \frac{d-n+t+k-s-1}{q-1}.\end{aligned}$$

The latter is equivalent to $t \le n - k +s - (d-q)/q$. $\square$

By applying the previous proposition to a set *S* of cardinality 1, we obtain the following result, which was presented in [14]. Note that the following bound can also be seen as a corollary of the Griesmer bound.

### Corollary 5.3

Let $\mathscr {C}\le \mathbb {F}_q^n$ of dimension $k \ge 2$ and minimum distance *d*. Then

$$\begin{aligned}d \le n - k +1 - (d-q)/q.\end{aligned}$$

Note the above result improves on the Singleton bound for $d \ge q$. Furthermore, it shows that for linear MDS codes one must have $d \le q$.

By applying Corollary 5.3 to the dual code $\mathscr {C}^{\perp }$ of an LRC $\mathscr {C}$, one can easily derive the following bound.

### Corollary 5.4

Let $\mathscr {C}\le \mathbb {F}_q^n$ with dimension $k \ge 2$, minimum distance *d*, and locality *r*. Moreover, let $d^\perp =d(\mathscr {C}^\perp )$. We have

$$\begin{aligned} d^\perp -1+\frac{d^\perp -q}{q} \le n-(d-2)-\left\lceil \llceil \frac{k}{r}\right\rceil \rrceil . \end{aligned}$$

### Proof

Note that if a code $\mathscr {C}$ has locality *r*, then by Lemma 3.1 there exists a codeword $x \in \mathscr {C}^\perp$ with $\omega ^{\text {H}}(x) \le r+1$. In particular, $d^\perp \le r+1$. Applying Corollary 5.3 we obtain

$$\begin{aligned} d^\perp \le k+1-(d^\perp -q)/q \end{aligned}$$

which, combined with Theorem 2.6, yields

$$\begin{aligned} d^\perp \le n-(d-2)-\left\lceil \llceil \frac{k}{r}\right\rceil \rrceil +1-(d^\perp -q)/q. \end{aligned}$$

This concludes the proof. $\square$

We now use the *generalized weights* of a code to derive new bounds which we later apply to LRCs. We start by recalling the needed definitions.

### Definition 5.5

Let $\mathscr {C}\le \mathbb {F}_q^n$ be a code. The *i*-**th generalized weight** of $\mathscr {C}$ is

$$\begin{aligned}d_i(\mathscr {C}):= \min \{|\sigma (\mathscr {D})| \,: \mathscr {D}\le \mathscr {C} and \dim (\mathscr {D}) = i\}\end{aligned}$$

for $1 \le i \le k$, and where $\sigma (\mathscr {D}):=\bigcup _{x \in \mathscr {D}} \sigma (x)$.

It is easy to see that a code $\mathscr {C}$ has minimum distance $d_1(\mathscr {C})$. Moreover, the generalized weights of a code are strictly increasing, i.e., $d_1(\mathscr {C})< d_2(\mathscr {C})< \ldots < d_k(\mathscr {C})$; see e.g. [33]. The following are well-known bounds involving the generalized weights of codes established respectively in [33] and [18, Theorem 1].

### Theorem 5.6

Let $\mathscr {C}\le \mathbb {F}_q^n$ be a code of dimension *k* and let $d_1, \ldots , d_k$ be its generalized weights. Then:

(i) $$d_i \le n - k + i$$,
(ii) $$(q^i -1)d_{i-1} \le (q^i -q)d_i$$.

Part (i) of Theorem 5.6 is often referred to as the *Singleton-type bound*.

The first two generalized weights of a code provide extremely useful information when trying to determine the smallest underlying field size over which some classes of codes exist. This is shown in the next result. It is, to the best of our knowledge, the first time such a bound is derived.

### Corollary 5.7

Let $\mathscr {C}\le \mathbb {F}_q^n$ be a code of minimum distance *d*. If $d_2 = d + s$, then

5.3 $$\begin{aligned} d \le sq. \end{aligned}$$

### Proof

By Theorem 5.6 (ii) we know that $(q^2 -1)d \le (q^2-q)d_2 = (q^2-q)(d+s)$. Rewriting this equality gives us $d \le sq$, as desired. $\square$

Note that Corollary 5.3 can also be established as a simple consequence of the previous theorem. In the context of Corollary 5.3, one may wonder if the bound of Proposition 5.2, when applied to a set *S* with $2 \le |S| \le k-1$, can lead to an upper bound for the |*S*|-generalized weight. In other words: Is it true that $d_{|S|} \le n- k+|S| - (d-q)/q$? Although there exist examples of codes for which the inequality holds, this is in general not the case, as illustrated by the following example.

### Example 5.8

Let $\mathscr {C}\le \mathbb {F}_2^7$ be the simplex code of dimension 3, i.e., the code generated by

$$\begin{aligned} G = \begin{pmatrix} 1 &  0 &  0 &  1 &  0 &  1 &  1 \\ 0 &  1 &  0 &  1 &  1 &  0 &  1 \\ 0 &  0 &  1 &  0 &  1 &  1 &  1 \end{pmatrix}.\end{aligned}$$

It is well known that $\omega ^{\text {H}}(x) = 4$ for all non-zero $x \in \mathscr {C}$. Therefore for all $S \subseteq [7]$ such that $|S| = 2$, the smallest weight of a codeword $x \in \mathscr {C}(S,[n])$ is 4. One can easily show that $d_2(\mathscr {C}) = 6$, which is greater than the right hand side of (5.1) when the parameters are those of the simplex code.

In the remaining part of this section, we study the generalized weights of an LRC. While these parameters were already considered in [16], we propose a new approach based on a new class of code parameters. The latter turn out to be closely related to the locality, and allow to provide concise proofs for known results and extend them as well.

### Notation 5.9

Let $\mathscr {C}\le \mathbb {F}_q^n$ be of dimension *k*, $\mathscr {C}^{\perp }$ its dual code of dimension $k^{\perp }=n-k$ with *i*-th generalized weight $d_i^{\perp }$. Define

$$\begin{aligned}\mu _i(\mathscr {C}):= \min \{t \,: \, d^{\perp }_t \ge n - k^{\perp } - (i-1) + t\}.\end{aligned}$$

The parameter $\mu _i(\mathscr {C})$ captures the smallest dimension *t* such that all subcodes of $\mathscr {C}^{\perp }$ of dimension *t* have support of size at least $n - k^{\perp } - (i-1) + t$. In other words, $\mu _i(\mathscr {C})$ is the smallest dimension of subcodes needed such that the generalized weight of the dual code of said dimension has defect at least $(i-1)$ from the Singleton-type bound.

### Remark 5.10

The parameters $\mu _i$ can be seen as a generalization of the parameter $\mu$ introduced in [32]. In the latter the parameter $\mu$ is defined in terms of matroid theory terminology but gives Notation 5.9 when translated into coding theory language.

Furthermore, the parameters $\mu _i$ can also be defined for matroids, however for readability purposes we restrict to defining those parameters in terms of coding theory terminology.

Before presenting the next result, we recall the following well-known facts; see e.g. [20].

### Lemma 5.11

Let $\mathscr {C}\le \mathbb {F}_q^n$ have dimension *k* and let $S \subseteq [n]$. Then

1. $$\dim (\mathscr {C}(S)) + \dim (\pi _{S^c}(\mathscr {C})) = k$$.
2. $$ \pi _S (\mathscr {C})^{\perp } = \pi _S (\mathscr {C}^{\perp }(S))$$

As the next result shows, the parameters $\mu _i$ are closely related to the generalized weights of the code $\mathscr {C}$. The following proof is inspired by the work of [32], in which similar methods were used to prove the same result for $i=1$.

### Theorem 5.12

Let $\mathscr {C}\le \mathbb {F}_q^n$ be a code of dimension *k* and let $d_i(\mathscr {C})$, for $1 \le i \le k$, be its generalized weights. Then

$$\begin{aligned}d_i(\mathscr {C}) = n - k - \mu _i(\mathscr {C}) + i +1.\end{aligned}$$

### Proof

For notation purposes, throughout the proof we let $\mu _i= \mu _i(\mathscr {C})$, $d_i = d_i(\mathscr {C})$, and $d_i^{\perp } = d_i(\mathscr {C}^{\perp })$. For ease of exposition, we divide the proof into two claims.

### Claim B

We have $$\mu _i \ge n - k - d_i +i +1$$.

### Proof of the claim

We show that $d^{\perp }_{k^{\perp } - d_i + i} < n-d_i+1$ by constructing a subspace of $\mathscr {C}^{\perp }$ that has the desired dimension and support. Let $\mathscr {D}\le \mathscr {C}$ and $S:= \sigma (\mathscr {D})$ with the property that $\dim (\mathscr {D}) = i$ and $|S| = d_i$. Since $\mathscr {D}$ achieves the generalized weight, it must be that $\mathscr {D}= \mathscr {C}(S)$ and by Lemma 5.11(1) we know that $\dim (\pi _{S^c}(\mathscr {C})) = k-i$. Furthermore, using Lemma 5.11(2) we obtain $\dim (\mathscr {C}^{\perp }(S^c)) = \dim (\pi _{S^c}(\mathscr {C})^{\perp }) = n - d_i - k + i$. Define $\mathscr {D}' = \mathscr {C}^{\perp }(S^c)$ and note that $|\sigma (\mathscr {D}')| \le n-d_i < n-d_i+1$. Therefore we have $d^{\perp }_{k^{\perp } - d_i +i} < n- d_i+1$, which establishes the statement.

### Claim C

We have $$\mu _i \le n - k - d_i + i + 1$$.

### Proof of the claim

First assume $\mu _i = 1$. From Theorem 5.6(1), we have $d_i \le n - k + i$ and therefore $\mu _i = 1 \le n-k-d_i + i +1$, as desired.

Now assume $\mu _i \ge 2$. Let $\mathscr {D}\le \mathscr {C}^{\perp }$ such that $\dim (\mathscr {D}) = \mu _i -1$ and $\sigma (\mathscr {D}) =: S$ satisfies $|S| = d^{\perp }_{\mu _i -1} \le k - i + \mu _i -1$. Such a subspace exists by definition of the parameter $\mu _i$. Furthermore, since $\dim (\mathscr {D}) = \mu _i -1$, $\sigma (\mathscr {D}) = S$ and $d_{\mu _i-1}^\perp = |S|$, we must have $\mathscr {D}= \mathscr {C}^{\perp }(S)$. Therefore using Lemma 5.11(2) we obtain:

$$\begin{aligned} \dim (\pi _S(\mathscr {C}))&= \dim (\pi _S(\mathscr {C}^{\perp }(S))^{\perp })\\&= |S| - \dim (\mathscr {C}^{\perp }(S)) \\&\le k -i + \mu _i - 1 - (\mu _i - 1)\\&= k-i. \end{aligned}$$

The above also gives that $|S| = \dim (\pi _S(\mathscr {C})) + \dim (\mathscr {D}) = \dim (\pi _S(\mathscr {C})) + \mu _i -1$, which will be used later on.

Using Lemma 5.11(1), we moreover get $s:= \dim (\mathscr {C}(S^c)) = k - \dim (\pi _S(\mathscr {C})) \ge i$. Let $G \in \mathbb {F}_q^{s \times n}$ be a generator matrix of $\mathscr {C}(S^c)$ in reduced row-echelon form.

Let $\mathscr {W} = \langle g_1, \ldots , g_i \rangle$, where $g_i$ is the *i*-th row of *G*. Clearly, $\dim (\mathscr {W}) = i$, $D:= \sigma (\mathscr {W}) \subseteq S^c$, and since *G* is in reduced row-echelon form, $\mathscr {C}(D) = \mathscr {W}$.

This shows that $d_i \le |D|$. Now consider $A \subseteq S^c$ such that $A \cup D = S^c$ and $A \cap D = \emptyset$. Note that $D^c = S \cup A$. By Lemma 5.11(1), we have $\dim (\pi _{A \cup S}(\mathscr {C})) = k - \dim (\mathscr {C}(D)) = k-i$. This leads to the following chain of inequalities:

$$\begin{aligned} k-i&= \dim (\pi _{A \cup S}(\mathscr {C}))\\&\le \dim (\pi _S(\mathscr {C})) + \dim (\pi _A(\mathscr {C})) \\&\le \dim (\pi _S(\mathscr {C})) + |A|. \end{aligned}$$

Finally, we use the facts that $d_i \le |D|$ and $|S| = \dim (\pi _S(\mathscr {C})) + \mu _i -1$ to get the following:

$$\begin{aligned} n- d_i&\ge n - |D|\\&= |S \cup A|\\&= |S| + |A|\\&\ge \dim (\pi _S(\mathscr {C})) + \mu _i -1 + k-i - \dim (\pi _S(\mathscr {C}))\\&= \mu _i + k -i -1. \end{aligned}$$

This shows that $\mu _i \le n- d_i - k +i+1$, as desired.

Combining Claim B with Claim C concludes the proof. $\square$

Our next move is to show how the dimension and locality of a code directly impact the parameter $\mu _i$. The special case of $\mu _1$ was already shown in [32].

### Lemma 5.13

Let $\mathscr {C}\le \mathbb {F}_q^n$ be an LRC of dimension *k*, locality *r*, generalized weights $d_i$, and $\mu _i$ as in Notation 5.9. Then for all $1 \le i \le k$ we have

$$\begin{aligned}\mu _i \ge \left\lceil \llceil \frac{k-(i-1)}{r} \right\rceil \rrceil .\end{aligned}$$

### Proof

Since $\mathscr {C}$ has locality *r*, there exist at least $\left\lceil \llceil k/r \right\rceil \rrceil -1$ linearly independent codewords $x_i \in \mathscr {C}^{\perp }$, for $1 \le i \le \left\lceil \llceil k/r \right\rceil \rrceil -1$, of weight at most $r+1$. Let $s:= \left\lceil \llceil (k - (i-1))/r \right\rceil \rrceil -1 \le \left\lceil \llceil k/r \right\rceil \rrceil -1$. Consider $\mathscr {D}= \langle x_1, \ldots , x_s \rangle$. Then

$$\begin{aligned} |\sigma (\mathscr {D})|&\le (r+1)\left( \left\lceil \llceil \frac{k-i+1}{r} \right\rceil \rrceil -1 \right) \\&\le k-i +1 +r -r + \left\lceil \llceil \frac{k-i+1}{r} \right\rceil \rrceil -1\\&< k- (i-1) + \left\lceil \llceil \frac{k-i+1}{r} \right\rceil \rrceil . \end{aligned}$$

Hence $\mu _i >\left\lceil \llceil (k- (i-1))/r \right\rceil \rrceil -1$, proving the lemma. $\square$

As a simple consequence we get the following characterization of optimal LRCs and a bound on the generalized weights of an LRC.

### Corollary 5.14

Let $\mathscr {C}\le \mathbb {F}_q^n$ be a code with dimension *k*, minimum distance *d* and locality *r*. Furthermore, let $\mathscr {C}^{\perp }$ be its dual code with generalized weight $\{d^{\perp }_1, \ldots , d^{\perp }_{n-k}\}$. The code $\mathscr {C}$ is an optimal LRC if and only if $d^{\perp }_{\lceil k/r\rceil } = n- k^{\perp } + \lceil k/r \rceil$.

### Proof

By Theorem 5.12, a code $\mathscr {C}$ is an optimal LRC if and only if $\mu _1 = \lceil k/r \rceil$. By definition, the latter is true if and only if $d^\perp _{\lceil k/r \rceil } \ge n - k^{\perp } + \lceil k/r \rceil$. However, by Theorem 5.6(i) we also know that $d^\perp _{\lceil k/r \rceil } \le n - k^{\perp } + \lceil k/r \rceil$. Hence equality must hold. $\square$

### Corollary 5.15

Let $\mathscr {C}\le \mathbb {F}_q^n$ be an LRC of dimension *k*, locality *r*, and let $d_i$ denote its generalized weights where $1 \le i \le k$. Then

$$\begin{aligned}d_i \le n -k +i - \left( \left\lceil \llceil \frac{k-(i-1)}{r} \right\rceil \rrceil -1\right) .\end{aligned}$$

### Proof

Combining Theorem 5.12 and Lemma 5.13 we obtain

$$\begin{aligned} d_i&= n-k - \mu _i +i+1 \\&\le n -k - \left\lceil \llceil \frac{k-(i-1)}{r} \right\rceil \rrceil +i +1. \end{aligned}$$

$$\square $$

### Remark 5.16

Theorem 5.15 is a special case of the bound established in [16, Theorem 1] for $(r, \delta )$-LRCs. However, the result therein is proved using the gap numbers of a code, which differs from our approach.

Using the Singleton-type bound (see Theorem 5.6 (i)), it is possible to determine or obtain a bound on the second generalized weight of optimal LRC. Note that in [16, Theorem 5], the authors establish the full generalized weight hierarchy of optimal LRCs under the assumption that *r*|*k*. The proof of [16, Theorem 5] is based on the following result, which we can prove for completeness using the theory developed in this paper.

### Proposition 5.17

Let $\mathscr {C}\le \mathbb {F}_q^n$ be an optimal LRC of dimension *k*, locality *r*, and let $d_i$ denote its *i*-th generalized weight, $1 \le i \le k$.

- If $$k \not \equiv 1 \pmod r$$, then $$d_2 = d_1 + 1$$.
- If $$k \equiv 1 \pmod r$$, then $$d_2 \le d_1 +2$$.

### Proof

Assume $k \not \equiv 1 \pmod r$. First note that $\left\lceil \llceil k / r \right\rceil \rrceil =\left\lceil \llceil (k-1) / r \right\rceil \rrceil$. Since $\mathscr {C}$ is optimal, we have $d = n - k - \left\lceil \llceil k / r \right\rceil \rrceil +2$. Using Theorem 5.15 we get the following bound on $d_2$:

$$\begin{aligned} d_2&\le n- k +2 - (\left\lceil \llceil (k-1) / r \right\rceil \rrceil ) + 1\\&\le n - k - \left\lceil \llceil k / r \right\rceil \rrceil + 3\\&= d_1 +1. \end{aligned}$$

However since $d_1 < d_2$ it must be that $d_2 = d_1 + 1$. Now assume $k \equiv 1 \pmod r$. Therefore $\left\lceil \llceil k / r \right\rceil \rrceil =\left\lceil \llceil (k-1) / r \right\rceil \rrceil +1$. Using Theorem 5.15 in a similar way as above, we get $d_2 \le d_1 + 2.$ $\square$

Combining Proposition 5.17 with Theorem 5.7 we get as an immediate corollary an upper bound on the minimum distance of optimal LRCs. A similar result was shown in [17, Theorem 2] by considering a suitable construction. We, on the other hand, show that the following bound can be seen as a direct consequence of the change in generalized weights of an optimal LRC.

### Corollary 5.18

Let $\mathscr {C}\le \mathbb {F}_q^n$ be an optimal LRC of dimension *k*, minimum distance *d*, and locality *r*.

- If $$k \not \equiv 1 \pmod r$$, then $$d \le q$$.
- If $$k \equiv 1 \pmod r$$, then $$d \le 2q$$.

### Remark 5.19

For $q=2$ there exist codes for which the bound in Theorem 5.18 for $k \equiv 1 \pmod r$ is attained. For example, the simplex code of Example 5.8 is an optimal LRC where $d = 4 = 2q$.

We can now derive a Singleton-type bound for LRCs that depends on the field size.

### Proposition 5.20

Let $\mathscr {C}\le \mathbb {F}_q^n$ be an LRC of dimension *k* and locality *r*. Then

5.4 $$\begin{aligned} d \le \frac{q}{q+1}(n - k - \left\lceil \llceil \frac{k-1}{r} \right\rceil \rrceil +3). \end{aligned}$$

### Proof

By Theorem 5.15 we have $d_2 \le n - k - \lceil \frac{k-1}{r} \rceil +3$. Hence $\smash {d_2 - d \le n - k - \left\lceil \llceil \frac{k-1}{r} \right\rceil \rrceil +3 - d}$ and applying Theorem 5.7 we get that

$$\begin{aligned}d \le q(n - k - \left\lceil \llceil \frac{k-1}{r} \right\rceil \rrceil +3 - d).\end{aligned}$$

By rewriting the inequality above we get the desired result. $\square$

Note that one can also derive Proposition 5.20 by combining Theorem 5.6 and Theorem 5.15. The bound of Proposition 5.20 outperforms the Singleton-type bound in (2.3) if $n-k-k/r+2 > q$ and *r* does not divide $k-1$, or if $n-k-k/r+2 > 2q$ and *r* divides $k-1$.

## Conclusion

In this paper, we introduced a refined notion of weight distribution for locally recoverable codes that captures both the locality and weights of codewords. This led to a duality result analogous to a MacWilliams identity, which, combined with linear programming, yields improved bounds on code parameters for several cases. We further analyzed the effect of field size using dual distances and generalized weights, obtaining new bounds and non-existence results for codes meeting the Generalized Singleton Bound over small fields.

Interesting questions remain open, as listed below.

(i) It is well known that Delsarte’s LP bound for classical codes implies other bounds that are more “explicit”, such as the Singleton and the Hamming bounds. These can be deduced by exhibiting feasible solutions of the dual program and applying weak duality in linear optimization. It would be interesting to investigate whether the LP bound introduced here can similarly lead to bounds that are weaker, but easier to evaluate than solving the linear program entirely.
(ii) A natural question arising from our results is whether a similar approach can be used to derive bounds for codes with availability, that is, codes in which each symbol can be recovered from several disjoint recovery sets.

## Data Availability

No datasets were generated or analysed during the current study.
